# The effect of phacoemulsification on visual function among Filipino cataract patients measured by a validated Filipino translation of Catquest-9SF

**DOI:** 10.1186/s12886-023-03072-3

**Published:** 2023-07-18

**Authors:** Geraldine Clare Marie P. Negre, Jose Ma. D. Martinez

**Affiliations:** East Avenue Medical Center, Quezon City, Metro Manila Philippines

**Keywords:** Cataract surgery, Catquest-8SF-PH, Catquest-9SF, Visual outcomes

## Abstract

**Background:**

This study developed a validated Filipino version of the Catquest-9SF and administered it to cataract patients pre- and post- surgery.

**Methods:**

This is a two-phase, single-center, cross sectional, questionnaire type study. The study included participants decked for surgery who are 18 years and above. Sampling was done purposively. The Catquest-9SF questionnaire was translated into Filipino, according to a standard procedure, and validated. The validated version was administered to the participants before and after unilateral cataract surgery. Data were analyzed using Rasch analysis.

**Results:**

Sixty-one patients were enrolled in the study. The preliminary Rasch analysis showed misfit of item 2, which was subsequently excluded from analysis. The remaining eight items showed person separation index of 2.70, reliability coefficient of 0.88, infit of 0.66 to 1.17, outfit of 0.66 to 1.49, observed raw variance explained by measures of 55.3% and eigenvalues of 1.9, 1.4, 1.2, 1.0 and 0.9. There was slightly poor targeting (mean person location 1.24) and multidimensionality but no evidence of differential item functioning (DIF). High internal consistency of items were observed (Cronbach’s alpha ≥ 90). Comparison of responses between pre- and post-surgery showed highly significant marginal homogeneity (*p* < 0.001).

**Conclusion:**

The Filipino translation of Catquest-9SF, the Catquest-8SF-PH, was highly valid. It showed improved perceived visual outcomes among Filipino patients post-cataract surgery.

## Background

In 2018, the Philippine National Survey of Blindness and Eye Disease Study Project [1] found that the major cause of visual impairment in the Philippines was cataract. It affected 1.112 million Filipinos. Cataract surgery is one of the top ten commonly performed surgeries [2] compensated by the Philippine Health Insurance Corporation (PHILHEALTH). Cataract therefore represents a significant disease burden with corresponding social and economic consequences.

Fortunately, visual improvement can be achieved in most cases by doing cataract surgery with lens implantation [3]. The measure of success in cataract surgery commonly focuses on quantitative outcomes like visual acuity and residual refraction [3]. These data describe the precision of the surgery acting as surrogate measures of success. Determining the impact of the surgery on the actual functioning of the patient is an equally important success measure [3]. Quantifying the benefit a patient receives from cataract surgery becomes very important in planning public health programs and health economics. Different questionnaires are employed to achieve this goal, such as the National Eye Institute Visual Functioning Questionnaire – 25 (NEI VFQ 25) [4], which measures important areas of well-being and functioning among patients with eye diseases. The NEI VFQ 25 has been translated into different languages, particularly Malay [5], Japanese [6] and Chinese [7] in Asia. An evaluation tool, specific for patients with cataract, that is widely used, translated, and adapted by different countries is the Catquest-9SF [8]. The short form version of the Catquest aimed to compare patient-reported visual function before and after cataract surgery. From 17 questions, it was trimmed down to nine using Rasch analysis. The study showed that Catquest-9SF was able to “measure disability, give an interval scoring scale, have high precision, minimize response burden, be sensitive to changes after cataract surgery, have high effect size and have good targeting” [8]. Given all these advantages from a clinical stand-point, this instrument has been translated into Spanish [9], Italian [10], Malay and Chinese [11], Chinese [12, 13], and Danish [14]. In Southeast Asia, however, particularly in the Philippines, no such translation and validation has been made.

There is no cataract registry in the Philippines, while other existing databases on cataract outcomes mostly focus on the objective aspects [15]. Organized datasets measuring subjective improvement, in terms of patient-reported outcomes, is non-existent in the Philippines. By this validated Filipino version of Catquest-9SF, Ophthalmologists in the Philippines will have a tool to measure the perceived visual disability of patients with cataract. This study aimed to determine the effect of phacoemulsification on the visual function and quality of life of Filipino cataract patients using a validated Filipino translation of the Catquest-9SF questionnaire.

## Methods

This was a two-phase, single-center, cross sectional, questionnaire type study, that was conducted at the eye center of a tertiary hospital from January to September 2019. It adhered to the basic principles of the Declaration of Helsinki and conformed with the guidelines set forth by the International Council for Harmonisation—Good Clinical Practice (ICH-GCP). The East Avenue Medical Center Institutional Ethics Review Board approved of the research project.

Participants were recruited purposively for this study. Sixty-one (61) patients for cataract surgery satisfying the inclusion/exclusion criteria were included in validating Catquest-9SF Filipino version to have 99% confidence that no item calibration was more than 1 logit away from its stable value.

The estimated sample size was computed to ensure that modelled item standard errors are in the range 2/√N < SE < 3/√N. Such minimum requirement also allows stratification of the sample by age or gender to check stability of item calibrations during analysis. This estimation is also higher than the minimum requirement needed to determine improvement in visual acuity after cataract surgery (n ≥ 16, computed using G*Power 3.1 [16], based on observed mean pre- and post-surgical Catquest scores of 0.32 ± 1.69 and -2.75 ± 2.17, respectively with effect size of 1.58 from the study Khadka J, et. al. in 2016 [12]).

Participants were recruited from the cataract surgery waiting list of the eye center between July to August 2019. Patients 18 years and above, with no severe cognitive impairment were included in the study. Ocular co-morbidities did not exclude them from participation. Patients unable to go through the informed consent process, mental or cognitive disabilities were excluded.

Participation of patients who cannot undergo cataract surgery but have previously answered the questionnaire was terminated. In cases of withdrawn participants, additional patients were recruited until the calculated sample size was reached as a lower sample size will affect the power of the study.

### Catquest-9SF Filipino version

The CATQUEST-9SF questionnaire was translated by 1) an Ophthalmologist and 2) a non-medical linguist into Filipino. The two versions were aligned and consolidated. A back-translation of the new version was done by a blinded translator to determine whether it was comparable to the original version.

Catquest-9SF Filipino version was tested on 10 patients with cataract randomly selected from the clinic to detect with high probability (> 80% power) at least one occurrence of the problem. The respondents were informed that they were in the testing phase. They were asked to assess the clarity of instructions with a score of 1–3: 1 as clear, 2 as somewhat clear and 3 as unclear. All items received a score of 1, hence there was no need to re-evaluate and rephrase. The questionnaire was then finalized and prepared for data collection to assess validity.

After the participants were provided with enough information about the questionnaire and cataract surgery, informed consent was obtained from them. The Filipino CATQUEST-9SF questionnaire was answered by cataract patients, either by self- or assistant administration. The questionnaire was then readministered one month after the cataract surgery.

### Statistical analysis

Summary statistics were reported as mean ± standard deviation for quantitative data with normal distribution or as median (interquartile range) for quantitative variables with non-normal distribution and as count (percent) for qualitative measures. Minimum and maximum values were also reported. Shapiro-Wilks test was used to check for normality assumption of continuous data. Rasch analysis was performed using pooled pre- and post-surgical data on patients with single-eye surgery for response category ordering, item fit statistics, principal components analysis, precision, differential item functioning and targeting. Person separation index, person-item map, infit mean square, principal components analysis of residuals and differential item functioning were used as bases for validity of the Catquest-9SF Filipino version. Cronbach’s α was also generated to measure internal consistency. Paired *t* test was used to compare Catquest scores before and after surgery. Statistical significance was based on *p*-value ≤ 0.05. STATA v14 and Winsteps v4.4.5 were used in data processing and analysis.

## Results

Of 61 patients who underwent cataract surgery, average age was 63 years, range from 20 to 81 years (Table 1). Majority were younger than 66 years (63.9%). Most patients were women (63.9%). Majority reached secondary level (38.2%). Hypertension (49.2%) and Diabetes mellitus (18%) were the most common comorbidities. Ocular co-morbidity was reported in 16.4%. About 26.2% underwent previous ocular surgery and 95.1% had uncomplicated surgery.

**Table 1:** Characteristics of patients who underwent cataract surgery

Characteristics	All Patients (*n* = 61)
Age in years	63 (12)
≤ 65	39 (63.9%)
≥ 66	22 (36.1%)
Gender	
Male	22 (36.1%)
Female	39 (63.9%)
Educational status	
Elementary	20 (36.4%)
High school	21 (38.2%)
College	12 (21.8%)
Post-graduate	2 (3.6%)
Comorbidities	
Diabetes mellitus	11 (18.0%)
Hypertension	30 (49.2%)
Stroke	3 (4.9%)
Others	8 (13.1%)
Phakic	
Both eyes	5 (8.8%)
Right eye	11 (19.3%)
Left eye	41 (71.9%)
Best corrected visual acuity in logMar	
Pre-surgery	1.3 ± 0.8
Post-surgery	0.1 ± 0.2
Laterality of operated eye	
Right	35 (57.4%)
Left	26 (42.6%)
Ocular comorbidity	
With	10 (16.4%)
Without	51 (83.6%)
Previous ocular surgery	
Yes	16 (26.2%)
No	45 (73.8%)
Intra/post-op complications	
With	3 (4.9%)
Without	48 (95.1%)

Data presented as mean ± standard deviation or count (percent)

Prior to surgery, majority of patients responded great difficulty to daily life activities in general (50.8%), reading text in the newspaper (42.6%), seeing to do handicrafts, woodwork, etc. (44.3%), seeing prices of goods when shopping (41%) and seeing to walk on uneven surfaces (41%). Most patients were generally dissatisfied with their vision (65.6%). Some patients had difficulty in recognizing faces of people they meet (39.3%).

After surgery, most patients reported no difficulty in daily life activities in general (63.9%) and all items related to the seven activities (55.7% to 78%). Majority were now satisfied with their vision (67.2%).

Comparison of responses between pre- and post-surgery showed highly significant marginal homogeneity (*p* < 0.001).

Rasch analysis was performed on all questionnaires and an exploratory analysis showed ordered probability curves, but a misfit of item 2 was observed based on infit and outfit mean square of 1.81. Therefore, this item was excluded in the analysis. Various translations and validations of the Catquest-9SF [12, 14] also had items removed, usually those pertaining to perceived difficulty in performing daily-life activities. The remaining 8 items had adequately fit the Rasch model.

The person separation index was 2.70 and reliability coefficient was 0.88 (Table 2), which corresponded to the ability of the questionnaire to differentiate among groups based on a 2.0 minimum separation index and 0.8 reliability.

**Table 2:** Summary statistics for person and item parameters of the Catquest-9SF Filipino version

Items	Measure	Separation Index	Reliability	Infit MNSQ	Outfit MNSQ
9-items	Person ability	2.77	0.88	1.00	1.03
	Item difficulty	3.13	0.91	1.02	1.03
8-items	Person ability	2.70	0.88	1.00	1.00
	Item difficulty	2.08	0.81	0.99	1.00

*MNSQ* Mean square error

**Fig. 1:**
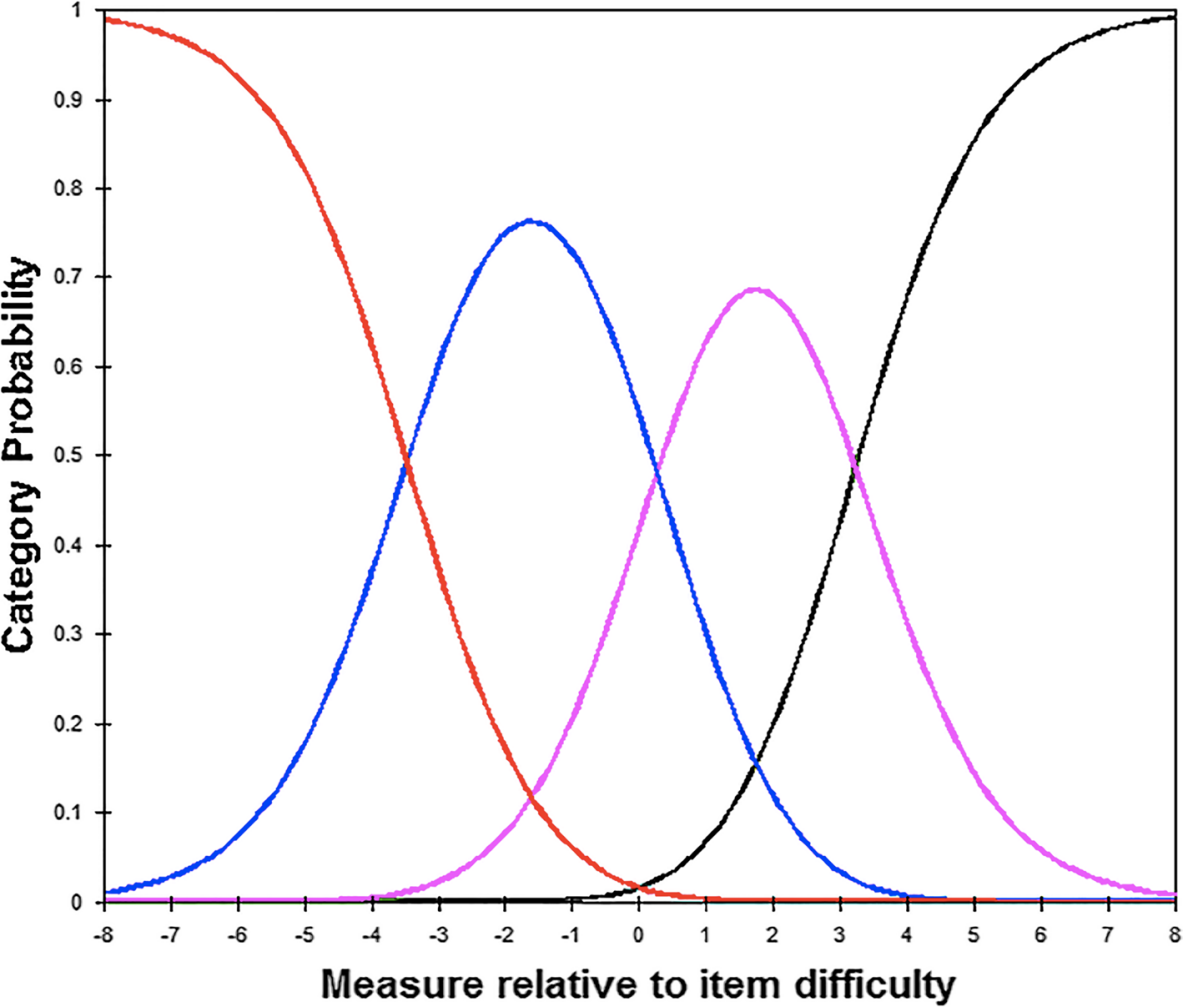
Category probability curves for the eight items in Catquest-8SF-PH

The response categories were ordered, and the category probability curves showed distinct response category thresholds (Fig. 1). This demonstrates that the participants answered logically. Category probability curves show the possibility of a category of being observed. There was no evidence of disordered thresholds.

The remaining 8 items showed an infit mean square from 0.66 to 1.17 and an outfit mean square from 0.66 to 1.49. Both fit statistics were within the range of 0.50 to 1.50, suggesting that the 8 items in the questionnaire were acceptable fit to the model (Table 3).

**Table 3:** Fit indices of the Catquest-8SF Filipino version

	Item	Item Calibration ± SE	Infit MNSQ	Outfit MNSQ
1	Daily-life activities in general	-0.08 ± 0.25	1.17	1.49
3	Reading text in the newspaper	-0.95 ± 0.25	1.13	1.14
4	Recognizing faces of people you meet	0.76 ± 0.2	1.24	1.19
5	Seeing prices of goods when shopping	-0.51 ± 0.25	0.88	0.81
6	Seeing to walk on uneven surfaces	-0.02 ± 0.25	0.77	0.73
7	Seeing to do handicrafts, woodwork, etc	0.28 ± 0.24	0.81	0.77
8	Reading subtitles on television	-0.32 ± 0.25	1.20	1.19
9	Seeing to engage in an activity/hobby	0.83 ± 0.25	0.66	0.66

*MNSQ* Mean square, *SE* Standard error

The principal components analysis showed that the observed raw variance explained by measures for empirical calculation was 55.3%, which was below 60%, suggesting evidence of multidimensionality. The unexpected variance explained by the first, second, third, fourth and fifth contrasts had eigenvalues of 1.9, 1.4, 1.2, 1.0 and 0.9, respectively. All contrasts were less than 2.0 eigenvalue units.

**Fig. 2:**
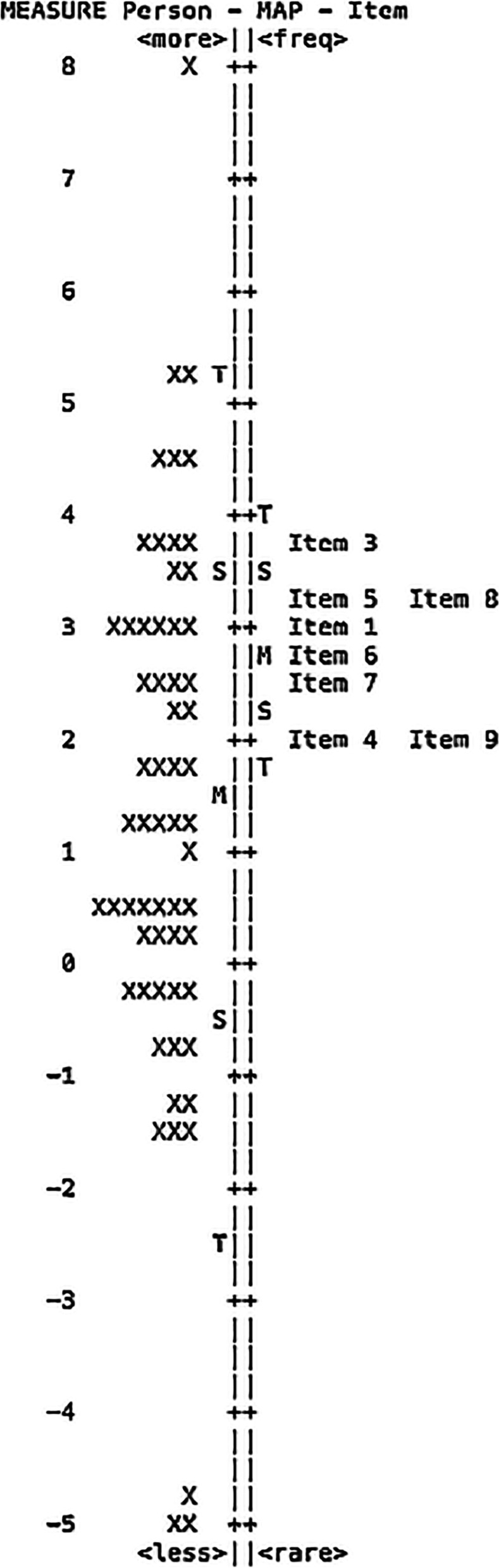
Person-item map of Catquest-8SF-PH presenting the distribution of Rasch-calibrated participant scores on the left and item locations on the right. (M = mean; S = one standard deviation; T = two standard deviations)

To determine whether the items were appropriate to the studied population of people with cataract, targeting precision was assessed by the pattern of the distribution appearing on the person-item map (Fig. 2) and by the difference in the value of person and item mean scores. There was an adequate spread of items across the range of person ability. However, the difference in person and item mean was 1.24 logits, suggesting significant mistargeting based on cut-off 1.0 logits. This mistargeting was also found in the Spanish, Danish and Chinese studies [9, 12–14]. Figure 2 likewise shows the relationship of the difficulty of the items on the questionnaire to the ability of patients. The easiest question was reading text in newspapers. The two most difficult questions were recognizing the faces of people you meet and seeing to engage in an activity/hobby that you are interested in.

There were no significant large differential item functioning for any of the items (more than 1.0 logits) across the different patient groups with values ranging from 0.00 to 0.6 logits (*p* > 0.05). Thus, the items were behaving similarly across the different subgroups of patients by age, gender, education, presence of comorbidity and complications.

Cronbach’s alpha (Table 4) of the Catquest-9SF Filipino version was 0.91 and 90 at pre- and post-surgery, respectively, which indicated excellent internal consistency, and did not change substantially even after deleting any of the items in the scale. The item-total correlation ranged from 0.576 to 0.799 at pre-surgery and from 0.597 to 0.794 at post-surgery, suggesting moderate to strong correlation or good homogeneity of the items for measuring patient-reported visual function.

**Table 4:** Internal consistency of the Catquest-9SF items

		Item-Total Correlation		Cronbach’s α if Item Deleted	
	Item	Pre-surgery	Post-surgery	Pre-surgery	Post-surgery
1	Daily-life activities in general	0.638	0.792	0.909	0.887
2	Satisfaction with vision	0.576	0.648	0.914	0.899
3	Reading text in the newspaper	0.701	0.710	0.905	0.894
4	Recognizing faces of people you meet	0.638	0.794	0.909	0.887
5	Seeing prices of goods when shopping	0.744	0.650	0.902	0.898
6	Seeing to walk on uneven surfaces	0.799	0.610	0.898	0.902
7	Seeing to do handicrafts, woodwork, etc	0.765	0.597	0.900	0.902
8	Reading subtitles on television	0.693	0.709	0.906	0.894
9	Seeing to engage in an activity/hobby	0.773	0.676	0.900	0.897
	Overall				
	9-items			0.914	0.906
	8-items			0.914	0.899

Comparison between pre- and post-surgery scores showed a significant decrease in scores post-operatively (0.77 vs. -2.30). Responsiveness to cataract surgery was very high based on effect size of 2.55, which was greater than the acceptable cut-off 1.0. This is higher compared to the overall effect size of 1.58 seen in the Chinese Catquest-8SF-CN [12]. The mean change of 3.07 logits is comparable to reports from Sweden [8] and Spain [9], with mean change of 3.5 and 3.9 logits respectively.

## Discussion

Rasch analysis of the Filipino version of Catquest-9SF showed misfit of item 2, satisfaction with current vision, which was subsequently excluded from analysis. With the 8 remaining items in the form, herein referred to as Catquest-8SF-PH, there was a slight decrease in person separation index from 2.77 to 2.70, but was still above the cut off value of 2.0. It is only in this study that a global question showed misfit. In the initial revision of the Catquest to Catquest-9SF, the seven disability questions formed the part with the most valid measurement, not the global questions [8]. A misfit item means that it measures something different than the overall scale. Visual disability is therefore not measured through satisfaction with vision among Filipino cataract patients. The reason for this may be because Filipinos have a generally positive attitude toward disability [17].

An instrument that is unidimensional means that it measures only one construct, in this case, visual disability. It is measured using three parameters [10] namely the fit statistics, principal components analysis of the residuals and the eigenvalue. Multidimensionality was seen in observed raw variance explained by measures for empirical calculation at 55.3%. However, the eigenvalues of unexpected variance explained by the first, second, third, fourth and fifth contrasts, met the cutoff value, proving unidimensionality.

The person-item map showed mistargeting of 1.24 logits. Inclusion of post-operative scores is the likely cause, likewise seen in other translation and validation studies [9, 12–14]. A ceiling or floor effect is seen when a large number of patients with no difficulty and high satisfaction [9] is included.

The mean pre-operative visual acuity was 1.3 log MAR (20/400). Post-operatively, it improved to a mean of 0.1 log MAR (20/25). With this objective improvement in vision there was an observed corresponding improvement in Catquest scores although a statistical correlation was not performed. Comparison of pre- and post-operative Catquest scores showed a large effect size of 2.55. Among the Catquest studies, the highest mean change was seen in Denmark (5.1 logits), which included bilateral cataract surgery participants.

Both the Catquest and the NEI VQF 25 prove to be useful tools in assessing visual function. Catquest, however, is shorter as it only has 9 questions, now trimmed down to 8 in the Filipino version.

The Catquest-8SF-PH’s limitation was that it did not discriminate among bilateral, first or second eye surgery. Perhaps an even larger effect may be seen if a longer interval from cataract surgery to post-surgical evaluation was observed to adequately correct near vision and any residual error of refraction. Another limitation was that the questionnaire was administered in only a single center in Metro Manila, which may not be representative of the entire Filipino population. Future administrations of the Catquest-8SF-PH could focus on identifying factors which lead to poor satisfaction. Comparison of intraocular lenses can likewise be done with this questionnaire.

## Conclusion

The translated Catquest-8SF-PH showed robust psychometric properties proving its validity in measuring visual disability in Filipino cataract patients. In its first application there was a significant improvement in the scores one-month post cataract surgery with a large effect size. Comparison among bilateral, first and second eye surgeries, long-term follow-up and administration in multiple centers in the Philippines are among the recommendations. The Catquest-8SF-PH is a short questionnaire that may be used to assess perceived visual disabilities among Filipino cataract patients and to measure success of surgical intervention.

## Data Availability

The datasets supporting the conclusions of this article are included within the article.
